# Lower Ultra-High Frequency Non-Deployable Omnidirectional Antenna for Nanosatellite Communication System

**DOI:** 10.3390/nano12183143

**Published:** 2022-09-10

**Authors:** Touhidul Alam, Muntasir M. Sheikh, Rabah W. Aldhaheri, Mandeep Singh Jit Singh, Mengu Cho, Mohammad Tariqul Islam, Khalid H. Alharbi, Md. Shabiul Islam

**Affiliations:** 1 Pusat Sains Ankasa (ANGKASA), Institut Perubahan Iklim, Universiti Kebangsaan Malaysia (UKM), Bangi 43600, Selangor, Malaysia; 2 Department of Computer Science and Engineering, International Islamic University Chittagong (IIUC), Kumira 4318, Bangladesh; 3 Department of Electrical and Computer Engineering, King Abdulaziz University, Jeddah 22254, Saudi Arabia; 4 Department of Electrical, Electronic and Systems Engineering, Faculty of Engineering and Built Environment, Universiti Kebangsaan Malaysia (UKM), Bangi 43600, Selangor, Malaysia; 5 Laboratory of Lean Satellite Enterprises and In-Orbit Experiments, Kyushu Institute of Technology, Fukuoka 804-8550, Japan; 6 Faculty of Engineering (FOE), Multimedia University, Persiaran Multimedia, Cyberjaya 63100, Selangor, Malaysia

**Keywords:** antenna, lower UHF, nanosatellite, omnidirectional

## Abstract

The concept of the nanosatellite comes into play in launching miniaturized versions of satellites or regarding payloads with minimizing cost and building time. The economic affordability of nanosatellites has been promoted with a view to launching various nanosatellite missions. The communication system is one of the most important aspects of a satellite. The antenna is a key element for establishing a communication link between the earth and the nanosatellite. The antenna and solar panel of the nanosatellite are two of the most vital components that profoundly impact antenna type and design. This paper proposes a non-deployable lower ultra-high frequency (UHF) antenna, strategically mounted on the satellite body, to address the constraints of deployment complexity and solar panel integration. The antenna was fabricated and performances measured with a 1U nanosatellite structure, which achieved resonance frequency at 401 MHz frequency bands with 0.672 dBi realized gain. The overall antenna size is 0.13λ × 0.13λ × 0.006λ. The major challenges addressed by the proposed antenna are to design a nanosatellite-compatible lower UHF antenna and to ensure solar irradiance into the solar panel to minimize input power scarcity.

## 1. Introduction

The development of nanosatellites or CubeSat provides a unique platform to explore space with a single unit structure of 10 × 10 × 10 cm^3^. Nowadays, numerous nanosatellite missions are launched into space for various applications, such as space education, space tethers, for commercial sectors and for remote sensing like weather forecasting, natural disaster monitoring, maritime tracking, and multispectral Earth imaging. Communication between satellite and earth is crucial for satellite communication, where an antenna plays a key role in the system. The operating frequency of the nanosatellite depends on its requirements and applications, and lower UHF are widely used bands in the nanosatellite communication system [1]. UHF antenna design is a challenging task for antenna researchers due to the inherent relation between antenna size and operating frequency, and nanosatellite standards [2]. Various antenna architectures have been studied and classified under two categories: non-deployable and deployable antennas [3]. Wire antennas, like monopoles and dipoles, are commonly used as the deployable antennas in the nanosatellite communication system. Deployment complexity might have a higher chance of satellite mission failure [4]. A 437 MHz half-wave crossed dipole antenna was developed for NUTS CubeSat [5], where element length was considered to be 172 mm. The antennas were made of measuring tape and wrapped around the satellite structure. A nichrome-wire deployment mechanism was utilized for deploying the antennas. To overcome this complexity, non-deployable antennas, like patch antennas, provide an effective solution with better mission reliability. In the last decade, various types of lower UHF patch antennas have been strategically integrated with the nanosatellite structure, designed with good efficiency and impedance bandwidth [5,6,7,8]. Mathur et al. developed a UHF patch antenna with high dielectric substrate material, which was designed for a 450 MHz USUSAT nanosatellite communication system [9]. In ref. [10], another printed patch antenna was developed for a UHF 433 MHz communication system, where −13 dB of gain was achieved using 51 × 28 mm^2^ FR-4 substrate material. In ref. [11], a folded shorted patch antenna was demonstrated for UHF 400 MHz microsatellite applications. The developed antenna offered CP performance with 130 mm × 130 mm ground plane.

Consequently, Podilchak et al. developed a multi-layered shorted patch antenna for UHF 400 MHz microsatellite applications [12]. The antenna achieved a gain of 0.4 dBiC with overall antenna dimensions of 150 mm × 150 mm × 37 mm. Metamaterial patch antennas have been explored for lower UHF nanosatellite communication systems [13,14]. However, these antennas occupy the surface space of the nanosatellite structure, constraining solar panel placement. Therefore, the conventional UHF patch antenna comes with larger space acquisition, and it becomes very challenging to mount adequate solar panels.

The transparent antenna is a potential antenna to overcome the complexity of having sufficient solar cells facing patch antennas, where the antenna is placed above the solar panels and integrated with the nanosatellite structure [15,16,17]. In [18], transparent antennas have been developed for ISM band CubeSat applications. However, for lower UHF antenna, the transparent antenna size becomes larger, making the design complex. In this case, the Planar Inverted F Antenna (PIFA) can overcome the problems associated with the antenna and solar panel placement on the limited surface of the nanosatellite structure [19,20,21]. In ref. [21], a modified PIFA antenna was developed for the microsat UHF communication system, where the PIFA structure occupied 85 × 85 × 31 mm^3^ space of the microsat structure. This paper presents a modified PIFA antenna for the 1U nanosatellite communication system. The metallic surface of the satellite body is considered as an infinite ground plane of the antenna. Moreover, the patch is tactically designed to pass solar irradiance into the solar panel.

## 2. Antenna Design

The antenna design process started by accumulating the design specification based on the UHF nanosatellite application and the commercially available 1U nanosatellite structure. The proposed antenna was designed considering the inverted F antenna technique to avoid the deployment complexity of the current nanosatellite antenna system [22,23]. The initial inverted F parameters and operating frequency were estimated using Equation (1), where *h* is the space between ground plane, *c* represents the velocity of light; and radiator patch, *L*1 and *L*2 are the patch length and height, respectively:

(1) $$f=\frac{c}{4\left(L1+L2+h\right)}$$

**Figure 1:**
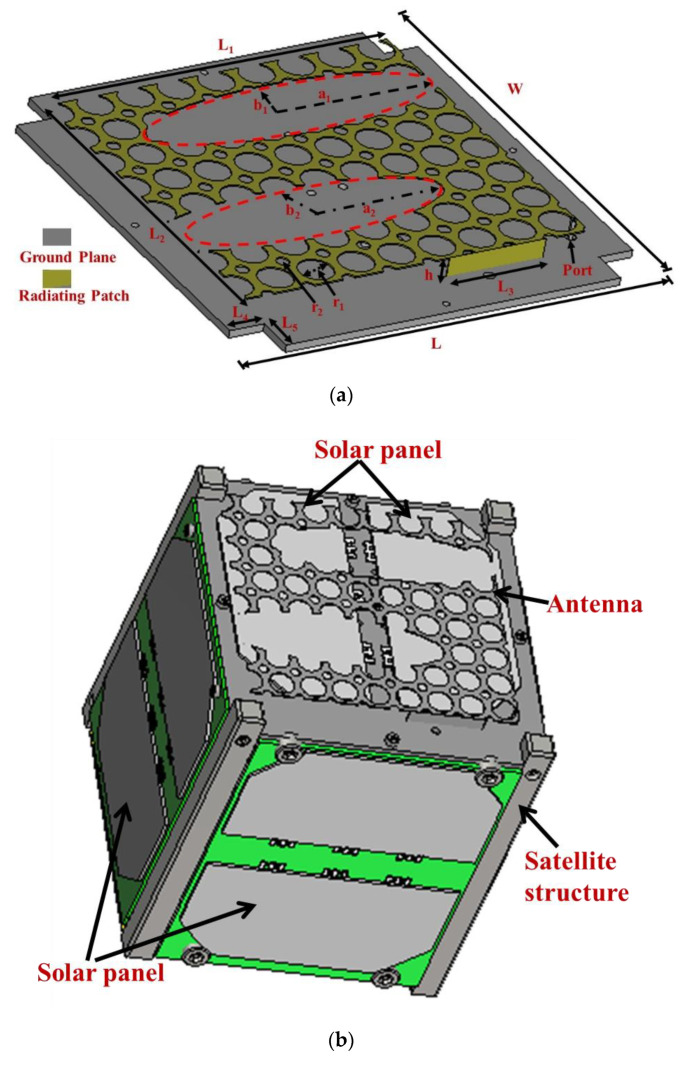
(**a**) Antenna geometry and (**b**) Antenna structure integrated with 1U nanosatellite in simulation environment.

The developed antenna addressed the inverse space accommodation relation between antenna and solar panel placement. The concept of the antenna over solar panels was adopted [24], and the antenna patch was modified to penetrate solar irradiance to the solar panel. The geometrical structure of the antenna was comprised of a rectangular-shaped patch with r_1_ and r_2_ net holes, two elliptical hollow spaces on the antenna patch, a shorting wall and a 50 Ω coaxial feed. The equivalent inductance introduced by the feeding probe and shorting wall was reduced by etching two elliptical slots and net holes. As a result, maximum uncovered surface area for sufficient solar penetration was achieved to mount the solar panel. Figure 1 depicts the antenna geometry and the final design parameters are tabulated in Table 1. The Japan Aerospace Exploration Agency (JAXA) standard was followed to design the 1U nanosatellite structure and the antenna mounted on the z-plane of the structure. A GaAs base solar panel was considered in the simulation.

## 3. Results and Discussions

**Figure 2:**
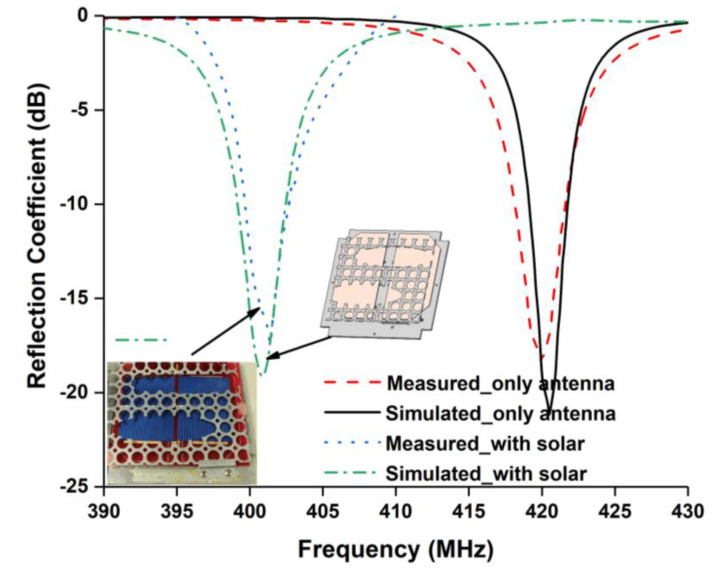
Reflection coefficient of the proposed antenna.

At first, the reflection coefficient of the proposed antenna was investigated and experimentally verified for an antenna without solar panels and with solar panel integration, as shown in Figure 2. It is shown in Figure 2 that the antenna operated at 420.5 MHz with −20.95 dB reflection coefficient before solar panel integration. The measured result showed −18.12 dB reflection coefficient at 419.8 MHz. The simulated and measured reflection bandwidths were 2.30 MHz (419.2–421.5 MHz) and 3.3 MHz (418.2–421.5 MHz), respectively. Both results were in good agreement. However, a little mismatch occurred due to fabrication tolerance. After that, the reflection coefficient was investigated with solar panel integration. Then the operating frequency shifted to 400.75 MHz due to the lossy material properties of the solar panel. The simulated and measured bandwidths were found to be 3.4 MHz (399.10–402.5 MHz) and 3.3 MHz (399.2–402.5 MHz), respectively.

**Figure 3:**
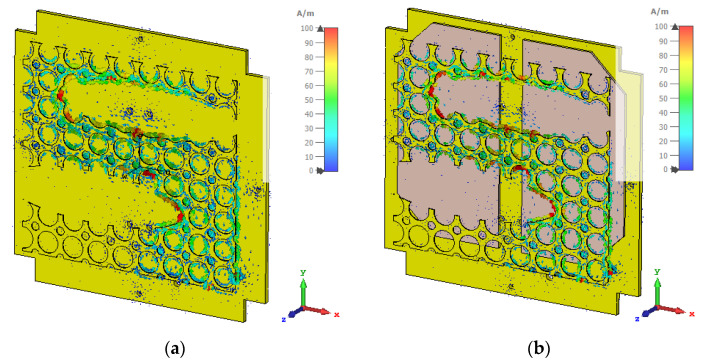
Surface current distribution of the proposed antenna—(**a**) only antenna and (**b**) antenna with solar panel.

The surface current distribution of the proposed antenna with and without solar panel integration was analyzed to understand the effective electrical length of the antenna. From Figure 3a, it is seen that the maximum current was observed near the shorting wall and the current flowed towards the elliptical slots. Moreover, some strong current was also observed at circular slots of the radiating patch. Therefore, a larger electric path was formed to miniaturize the antenna structure, and about 60% of the surface was truncated for solar irradiance. Figure 3b also shows a similar pattern after solar panel integration.

**Figure 4:**
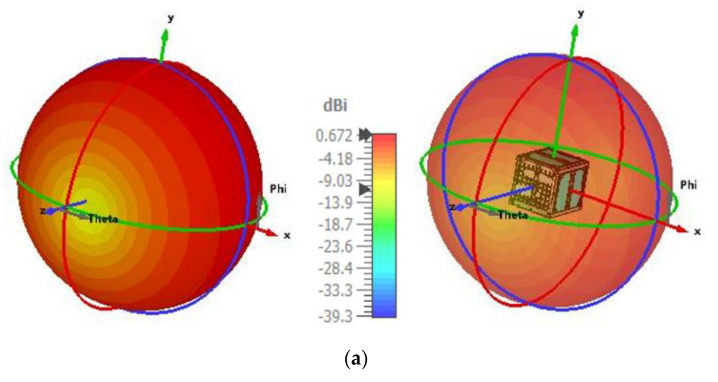

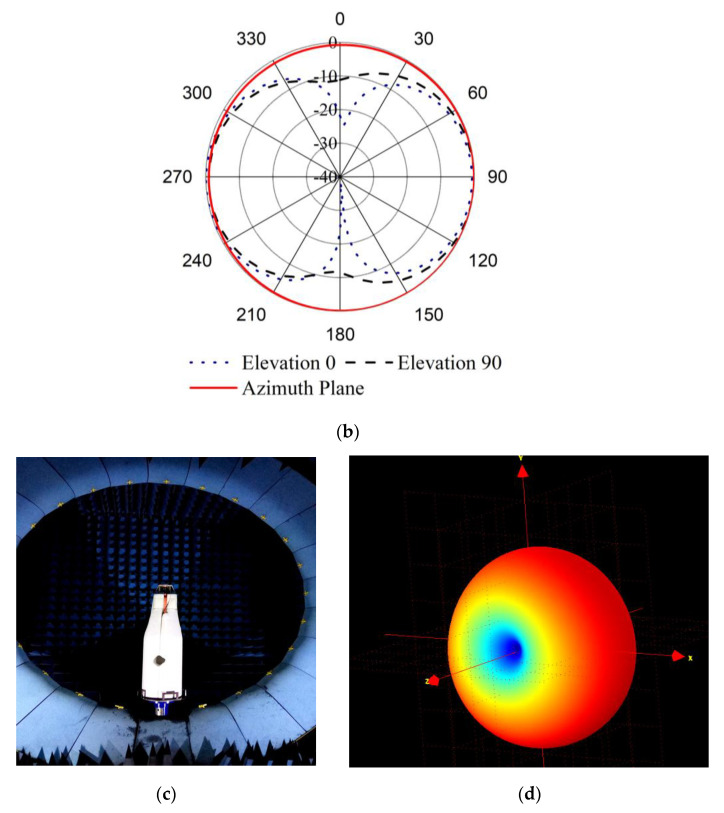
Antenna radiation pattern with satellite structure—(**a**) simulated 3D radiation pattern, (**b**) 2D radiation pattern (**c**) measurement setup and (**d**) measured radiation pattern.

**Figure 5:**
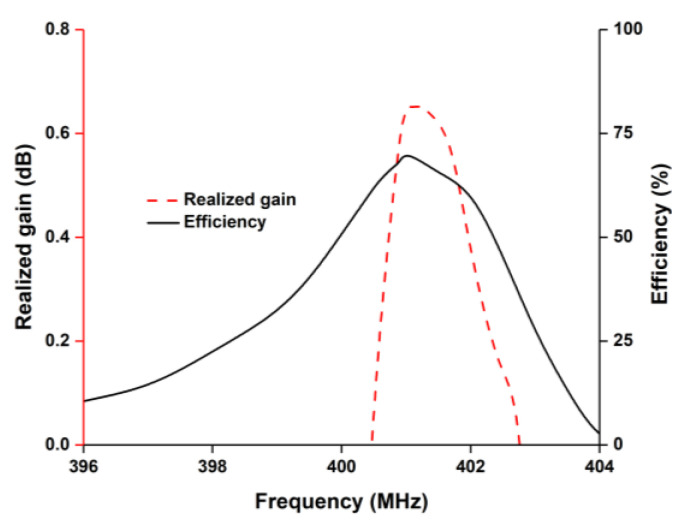
Antenna radiation efficiency and gain pattern with satellite structure.

The radiation pattern of the proposed antenna was analyzed with 1U nanosatellite structure. Both 3D and the 2D radiation patterns are presented in Figure 4a,b. The antenna achieved 0.672 dB of realized gain at the 401 MHz frequency band, as seen in Figure 4a. Figure 4b shows that the antenna achieved a nearly omnidirectional radiation pattern in the azimuth plane. The radiation characteristics of the antennas were measured in Satimo’s Star Lab near the field antenna measurement system at Laird Technologies (M) Sdn Bhd, Malaysia, as shown in Figure 4c. The simulation and measured radiation patterns showed a little discrepancy. This discrepancy arose out of the fact that the nanosatellite model used in the simulation and the fabricated model used in measurement were not exactly equivalent, due to differences in material properties of the nanosatellite structure. The antenna showed 70.88% of total efficiency and 0.672 dB realized gain with 1U nanosatellite structure, as shown in Figure 5.

**Figure 6:**
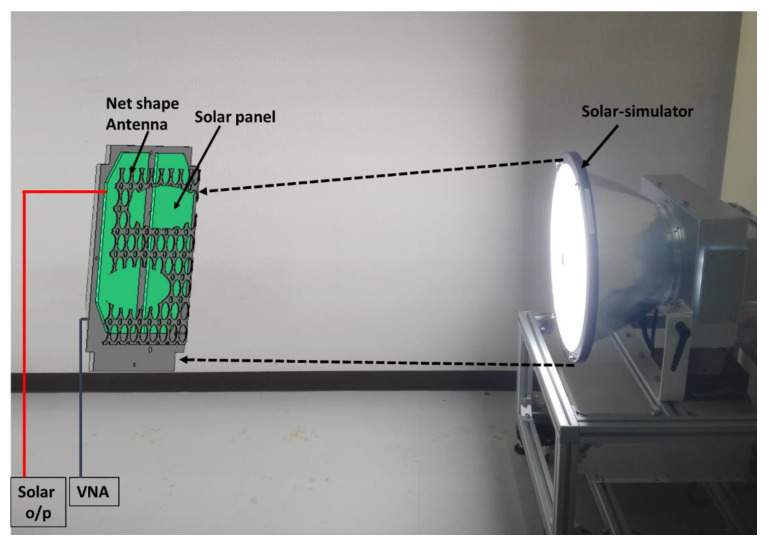
Solar power output investigation with antenna structure integration.

The solar panel output power with and without antenna integration was also investigated, shown in Figure 6. Solar simulator SML-2K1MV1 and pyranometer MS-802 were used in this measurement. Initially, the solar panels were placed satellite backplane board and output power was measured. Then, the antenna was placed upon the solar panel and solar panel output power was measured. The structural shadow effects were investigated for 0°, 45° and 60° solar panel positions with respect to the simulator. The results are presented in Table 2.

A performance comparison with different types of recent UHF antennas is presented in Table 3. Most antennas suffer from different limitations, like larger size, occupied solar panel space and design complexity. Therefore, the proposed antenna facilitates a substantial trade-off between antenna performances and the size of 1U nanosatellite communication constraints.

## 4. Conclusions

This paper presents an omnidirectional lower UHF band antenna for the 1U nanosatellite communication system, which is highly reliable, with a simplified structure of 0.13λ × 0.13λ × 0.006λ. The antenna is free of deployment complexity and facilitates sufficient space for solar panel placement. An omnidirectional radiation pattern, 0.672 dB realized gain and verified measured results are the features of this antenna. Therefore, the proposed antenna is a potential solution for deployment free nanosatellite communication and allows satellite engineers to focus on other design criteria.

## Figures and Tables

**Table 1:** Design parameters of the proposed antenna.

Parameters	Value (mm)	Parameters	Value (mm)
L	100	L_4_	8.5
W	100	L_5_	8.5
h	5	a_1_	36
L_1_	86	a_2_	40
L_2_	78	b_1_	10
L_3_	24	b_2_	7
r_1_	4	r_2_	1.5

**Table 2:** Solar panel output power investigation of the proposed antenna.

Condition	Solar Panel Rotation (Deg)	Solar Panel Output Power (W)	Effective Power (%)
Normal Solar panel	0°	0.926	100
Solar power integrated with Antenna structure	0°	0.885	95.57
	45°	0.663	71.60
	60°	0.625	67.5

**Table 3:** Comparison between proposed antenna and existing lower UHF antennas.

Antenna	Size (mm)	Operating Frequency (MHz)	Gain (dB)	Remarks
Folded Microstrip antenna [12]	150 × 150 × 37	384–410	0.4	Larger size and incompatible with 1U nanosatellite
Patch antenna [14]	80 × 45 × 1.575	391–405.92	1.77	Compatible with 1U nanosatellite but occupied solar panel space
Patch antenna [13]	80 × 40 × 3.35	443.5–455	2.5	Compatible with 1U nanosatellite but occupied solar panel space
Modified PIFA [19]	80 × 90 × 6	447.5–453.5	0.6	Solar panel integrated with 1U nanosatellite
Inverted-F antenna [21]	85 × 85 × 31	401.8	5.37	High gain but incompatible with 1U nanosatellite
Meander line patch [25]	50 × 80 × 1.635	920	1.8	Compatible with 1U nanosatellite but occupied solar panel space
Proposed (Modified PIFA)	78 × 86 × 5	399.2–402.5	0.672	Solar panel integrated with 1U nanosatellite

## Data Availability

The data presented in this study are presented in this article.

## References

[1] Kulu E. Nanosats Database|Constellations, Companies, Technologies and More. https://www.nanosats.eu/.

[2] Rahmat-Samii Y., Manohar V., Kovitz J.M. (2017). For Satellites, Think Small, Dream Big: A review of recent antenna developments for CubeSats. IEEE Antennas Propag. Mag..

[3] Gao S., Rahmat-Samii Y., Hodges R.E., Yang X.-X. (2018). Advanced Antennas for Small Satellites. Proc. IEEE.

[4] Ernest A.J., Tawk Y., Costantine J., Christodoulou C.G. (2014). A Bottom Fed Deployable Conical Log Spiral Antenna Design for CubeSat. IEEE Trans. Antennas Propag..

[5] Fujishige T., Ohta A., Tamamoto M., Goshi D., Murakami B., Akagi J., Shiroma W. Active antennas for cubesat applications. Proceedings of the 16th AIAA/USU Annual Small Satellites Conference.

[6] Kakoyiannis C., Constantinou P. (2011). Electrically small microstrip antennas targeting miniaturized satellites: The cubesat paradigm. Microstrip Antennas.

[7] Islam M.T., Cho M., Samsuzzaman M., Kibria S. (2015). Compact Antenna for Small Satellite Applications [Antenna Applications Corner]. IEEE Antennas Propag. Mag..

[8] Samsuzzaman M., Islam M.T., Kibria S., Cho M. (2018). BIRDS-1 CubeSat Constellation Using Compact UHF Patch Antenna. IEEE Access.

[9] Mathur R., Haupt R., Swenson C. Student antenna design for a nanosatellite. Proceedings of the 2001 IEEE Aerospace Conference Proceedings (Cat. No. 01TH8542).

[10] Buckley J., Gaetano D., McCarthy K., Loizou L., O’Flynn B., O’Mathuna C. (2014). Compact 433 MHz antenna for wireless smart system applications. Electron. Lett..

[11] Podilchak S.K., Caillet M., Lee D., Antar Y.M.M., Chu L., Cain J., Hammar M., Caldwell D., Barron E. Compact antenna for microsatellite using folded shorted patches and an integrated feeding network. Proceedings of the 2012 6th European Conference on Antennas and Propagation (EUCAP).

[12] Podilchak S.K., Murdoch A.P., Antar Y.M. (2017). Compact, Microstrip-Based Folded-Shorted Patches: PCB antennas for use on microsatellites. IEEE Antennas Propag. Mag..

[13] Alam T., Almutairi A.F., Samsuzzaman M., Cho M., Islam M.T. (2021). Metamaterial array based meander line planar antenna for cube satellite communication. Sci. Rep..

[14] Alam T., Islam M.T., Cho M. (2019). Near-zero metamaterial inspired UHF antenna for nanosatellite communication system. Sci. Rep..

[15] Nashad F., Foti S., Smith D., Elsdon M., Yurduseven O. Development of transparent patch antenna element integrated with solar cells for Ku-band satellite applications. Proceedings of the 2016 Loughborough Antennas & Propagation Conference (LAPC).

[16] Selamat A., Misran N., Mansor M.F., Islam M.T., Zaidi S.H. (2014). Scattering Microwave Signal Analysis from Triangular Loop Element of Different Transparent Conductive Thin Films at Ku-band (Analisis Penyerakan Isyarat Gelombang Mikro Elemen Gelung Segi Tiga dari Pelbagai Jenis Filem Pengalir Lutsinar pada Jalur-Ku). J. Kejuruter..

[17] Paul L.C., Pramanik R.K., Rashid M.U., Sarker S., Mahmud Z., Islam M.T. An ITO Based High Gain Optically Transparent Wide Band Microstrip Antenna for K Band Satellite Communication. Proceedings of the 2019 International Conference on Robotics, Electrical and Signal Processing Techniques (ICREST).

[18] Liu X., Jackson D.R., Chen J., Liu J., Fink P.W., Lin G.Y., Neveu N. (2017). Transparent and Nontransparent Microstrip Antennas on a CubeSat: Novel low-profile antennas for CubeSats improve mission reliability. IEEE Antennas Propag. Mag..

[19] Alam T., Islam M.T., Ullah M.A., Cho M. (2018). A Solar Panel-Integrated Modified Planner Inverted F Antenna for Low Earth Orbit Remote Sensing Nanosatellite Communication System. Sensors.

[20] Alam T., Islam M.T., Ullah A., Rahmatillah R., Aheieva K., Lap C.C., Cho M. (2018). Design and compatibility analysis of a solar panel integrated UHF antenna for nanosatellite space mission. PLoS ONE.

[21] Hu H., Liao W., Hou L., Sun F., Zhang G., Ren C., Zhang X. (2020). Compact Planar Inverted-F Antenna for MicroSats Omnidirectional Communications. IEEE Antennas Wirel. Propag. Lett..

[22] Haruki H. (1982). The inverted-F antenna for portable radio units. Conv. Rec. IECE Japan Mar..

[23] Tsunoda K., Taga T. Analysis of planar inverted F antenna using spatial network method. Proceedings of the International Symposium on Antennas and Propagation Society, Merging Technologies for the 90’s.

[24] Yekan T., Baktur R. (2017). Conformal Integrated Solar Panel Antennas: Two effective integration methods of antennas with solar cells. IEEE Antennas Propag. Mag..

[25] Zalfani N., Beldi S., Lahouar S., Besbes K. (2021). Miniaturized Planar Meander Line Antenna for UHF CubeSat Communication. Adv. Space Res..

